# Pulmonary cryptococcosis in routine care: the clinical–radiologic spectrum and transparent evidence-tier reporting (confirmed versus presumed) among patients with no recorded immunocompromising conditions

**DOI:** 10.1099/jmm.0.002159

**Published:** 2026-04-22

**Authors:** Xiao-yao Zhang, Hui Ding, Li-hong Pei, Qing-ping Ye, Wei-bo You, Jian-min Ren

**Affiliations:** 1 Department of Clinical Laboratory, Lishui Municipal Central Hospital (Lishui Hospital Zhejiang University), Lishui, Zhejiang, PR China

**Keywords:** chest computed tomography, cryptococcal antigen, evidence tier, immunocompromise, pulmonary cryptococcosis, real-world cohort

## Abstract

**Introduction.** Pulmonary cryptococcosis is increasingly identified in routine care, including among patients with no recorded immunocompromising conditions.

**Hypothesis/Gap Statement.** Real-world descriptions of pulmonary cryptococcosis are difficult to interpret because diagnostic intensity and case definitions vary across studies, and many cohorts reflect pathway-based rather than population-based case capture.

**Aim.** This study aimed to describe the clinical presentation, including asymptomatic/incidental cases, and chest computed tomography (CT) patterns of pulmonary cryptococcosis by two-level immune status, and to report prespecified diagnostic evidence tiers (confirmed vs presumed).

**Methodology.** We conducted a single-centre, retrospective, observational study at a tertiary hospital in China (January 2016 to December 2020). The analytic cohort comprised adjudicated pulmonary cryptococcosis cases identified along a routine-care diagnostic pathway among patients with pulmonary imaging abnormalities who underwent serum cryptococcal antigen testing as part of clinical evaluation. Cases were classified using a prespecified two-tier evidence scheme (confirmed vs presumed).

**Results.** Among 62 cases, 23/62 (37.1%) were immunocompromised, and 39/62 (62.9%) had no recorded immunocompromising conditions. Evidence tiers comprised 32/62 (51.6%) confirmed and 30/62 (48.4%) presumed cases. Cryptococcal meningitis was documented in 11/62 (17.7%). Asymptomatic/incidental presentation was recorded in 16/62 (25.8%), including 4/23 (17.4%) immunocompromised cases and 12/39 (30.8%) cases with no recorded immunocompromising conditions. On chest CT, nodules/masses were the most frequently recorded pattern in both groups, observed in 17/23 (73.9%) and 31/39 (79.5%) cases, respectively. Opacities/consolidation, cavitation and loculated pleural effusion were recorded less often.

**Conclusion.** In this routine-care diagnostic-pathway cohort, nodules/masses were the most frequently recorded CT pattern, and asymptomatic/incidental presentation was documented in a substantial proportion of cases, including among patients with no recorded immunocompromising conditions. Separate reporting of confirmed and presumed cases may improve interpretation of cohorts assembled under non-uniform diagnostic work-up.

## Data Summary

The datasets generated and/or analysed during the current study are not publicly available due to patient privacy and institutional data governance restrictions, but are available from the corresponding author on reasonable request and with approval from the Institutional Ethics Committee, where applicable.

## Introduction

Pulmonary cryptococcosis is an important fungal infection with a broad clinical spectrum, ranging from incidental pulmonary nodules to severe disseminated disease, and its presentation is shaped by host factors and the diagnostic intensity applied in routine care [1]. Although historically regarded as predominantly affecting immunocompromised hosts, pulmonary cryptococcosis is increasingly identified among patients with no recorded immunocompromising conditions, often with mild or absent symptoms and imaging findings that overlap with malignancy or other granulomatous diseases [1,2]. On chest computed tomography (CT), pulmonary cryptococcosis may present as nodules or mass-like lesions – particularly among patients with no recorded immunocompromising conditions – creating diagnostic uncertainty and often prompting invasive sampling in routine practice [2,3].

Serum cryptococcal antigen (CrAg) testing is well established in the diagnostic work-up of cryptococcal meningitis and disseminated cryptococcosis and shows strong analytical performance in modern lateral flow formats [4,5]. In pulmonary cryptococcosis, however, serum CrAg positivity and its clinical correlates vary with immune background, disease burden and extent of involvement. Prior cohort studies have described associations between serum CrAg and clinical manifestations, CT features and immune status, but many have used heterogeneous case definitions and non-uniform diagnostic work-up patterns [6,10]. Because imaging-detected pulmonary nodules are a common entry point to evaluation, distinguishing infectious from neoplastic aetiologies remains challenging, and imaging clues have recognized limitations even in specialized populations [11,12].

At our institution, serum CrAg testing is widely available and is frequently ordered during evaluation of pulmonary imaging abnormalities as part of routine care. In this setting, pulmonary cryptococcosis is encountered along a CrAg-based diagnostic pathway, but published real-world descriptions remain difficult to compare because diagnostic intensity and case definitions vary across studies [1,2, 6,10]. We therefore assembled a retrospective diagnostic-pathway cohort of adjudicated pulmonary cryptococcosis cases identified among CrAg-tested patients undergoing evaluation for pulmonary imaging abnormalities, and aimed to characterize presenting symptoms, including asymptomatic/incidental cases, and chest CT patterns; to describe these phenotypes by two-level immune status; and to report a prespecified evidence-tier classification (confirmed vs presumed) alongside denominators for key diagnostic procedures.

## Methods

### Study design, setting and study period

This was a single-centre, retrospective, observational study conducted at Lishui Municipal Central Hospital, China. The study period was 1 January 2016 to 31 December 2020; follow-up was administratively censored on 31 December 2020.

### Data sources and data extraction

We retrospectively extracted data by linking the Hospital Information System (HIS) encounters, Laboratory Information System (LIS) results, radiology/Picture Archiving and Communication System (PACS) reports and pathology records within the electronic medical record system. We extracted demographics, underlying diseases and immune status, presenting symptoms, imaging findings, serum CrAg results, downstream diagnostic procedures (including confirmatory tests and tissue sampling), treatment strategies and follow-up outcomes.

### Source population and analytic sampling frame

The source population comprised patients who underwent diagnostic evaluation at our hospital during the study period because of pulmonary imaging abnormalities (e.g. opacities/consolidation or nodules). Because a retrospective registry of all patients with suspected pulmonary cryptococcosis was not available, we used a serum CrAg test ordered during these evaluations as a traceable marker of entry into the routine-care diagnostic pathway.

The analytic sampling frame was restricted to patients who (i) attended Lishui Municipal Central Hospital between January 2016 and December 2020; (ii) had chest imaging that reported pulmonary opacities/consolidation or nodules; and (iii) underwent serum CrAg testing as part of the clinical evaluation. This study therefore characterizes adjudicated pulmonary cryptococcosis cases identified along a local routine-care diagnostic pathway among CrAg-tested patients with pulmonary abnormalities, rather than all patients with pulmonary imaging abnormalities or all pulmonary cryptococcosis cases in the underlying population.

Patients were excluded if available clinical or imaging data were insufficient for basic case adjudication or if follow-up information within the hospital record was unavailable for classification of downstream diagnostic procedures and outcomes.

### From the sampling frame to the final case cohort

Patients who met the sampling-frame criteria comprised the screened population. From this screened population, we constructed the final analytic case cohort by identifying patients adjudicated as pulmonary cryptococcosis and classifying diagnostic certainty using prespecified evidence tiers (R1; confirmed vs presumed). Only adjudicated pulmonary cryptococcosis cases were included in phenotype summaries and between-group descriptive comparisons.

### Index date, follow-up window and de-duplication

The index date was defined as the date of the first (index) serum CrAg test during the study period. The follow-up window extended from the index date to the last clinical record available within the hospital system, with administrative censoring on 31 December 2020.

We analysed data at the patient/episode level to prevent duplicate counting. When multiple encounters or repeated CrAg tests occurred for the same patient, only the first eligible episode (index episode) was retained for the primary cohort. For patients with multiple eligible records on the same index date, we prioritized the record with the most definitive evidence (culture/pathology) for evidence-tier assignment; the remaining duplicates were excluded.

### Diagnostic testing coverage

We report key test denominators to make diagnostic intensity explicit across the final case cohort: respiratory culture was performed in 62/62 cases, serum CrAg was tested in 62/62, lung biopsy/needle aspiration with fungal culture and special stains was performed in 21/62, and lumbar puncture with cerebrospinal fluid (CSF) culture plus CSF CrAg testing was performed in 15/62.

### Evidence-tier classification

We prespecified a two-tier evidence scheme (R1) and reported evidence tiers separately throughout. In this cohort, CSF-based testing (lumbar puncture with CSF culture and CSF CrAg testing) was available in 15/62 cases, and lung biopsy/needle aspiration with fungal culture and special stains was available in 21/62, reflecting selective verification in routine care.

***Confirmed pulmonary cryptococcosis*** was defined by at least one of the following:

1. positive fungal culture from a respiratory specimen or a sterile site, and/or
2. histopathology and/or special stains from a pulmonary specimen consistent with cryptococcosis.

***Presumed pulmonary cryptococcosis*** was assigned using a prespecified adjudication algorithm and required all of the following:

1. no microbiological or pathological confirmation of cryptococcosis identified in the available record;
2. a compatible clinical–radiologic pulmonary presentation;
3. positive serum CrAg as supportive evidence;
4. review of the available record for major alternative explanations, such as malignancy, tuberculosis, other fungal infection, other pulmonary infection or noninfectious inflammatory disease; and
5. no more plausible alternative diagnosis retained after clinician adjudication based on the available record.

Treatment response was not used as a standalone diagnostic criterion, and serum CrAg positivity alone was not considered sufficient for case classification. Because invasive diagnostic procedures were performed selectively in routine care, confirmed cases were more likely to occur among patients who underwent pathology-based or other higher intensity verification.

### Immune status classification

For the primary analysis, immune status was categorized into two levels (immunocompromised vs no recorded immunocompromising conditions) based on chart review of major host factors and immunosuppressive therapies. For supplementary description, we further stratified cases into three levels (major immunosuppression, comorbidity-associated vulnerability and no recorded immunocompromising conditions) and presented these results descriptively due to sparse cells.

### Outcomes and variables

We summarized clinical presentation, imaging patterns, downstream diagnostic procedures, treatment and follow-up outcomes. Evidence of central nervous system (CNS) dissemination was summarized descriptively based on documented cryptococcal meningitis and/or CSF-based evidence consistent with CNS involvement, when available.

### Statistical analysis

All analyses were descriptive. Continuous variables were presented as median [interquartile range (IQR)] and categorical variables as *n* (%). Clinical presentation, chest CT patterns, evidence tiers, diagnostic testing coverage and immune-strata summaries were described using prespecified denominators. Because this was a single-centre, retrospective, diagnostic-pathway cohort with selective verification and sparse subgroup cells, the analyses were not used to support formal between-group inference. Symptom summaries (Table S1, available in the online Supplementary Material), chest CT feature distributions (Table S2) and three-level immune-strata summaries (Fig. S1) were therefore presented descriptively without hypothesis testing.

## Results

### Cohort overview and immune status distribution

**Fig. 1.**
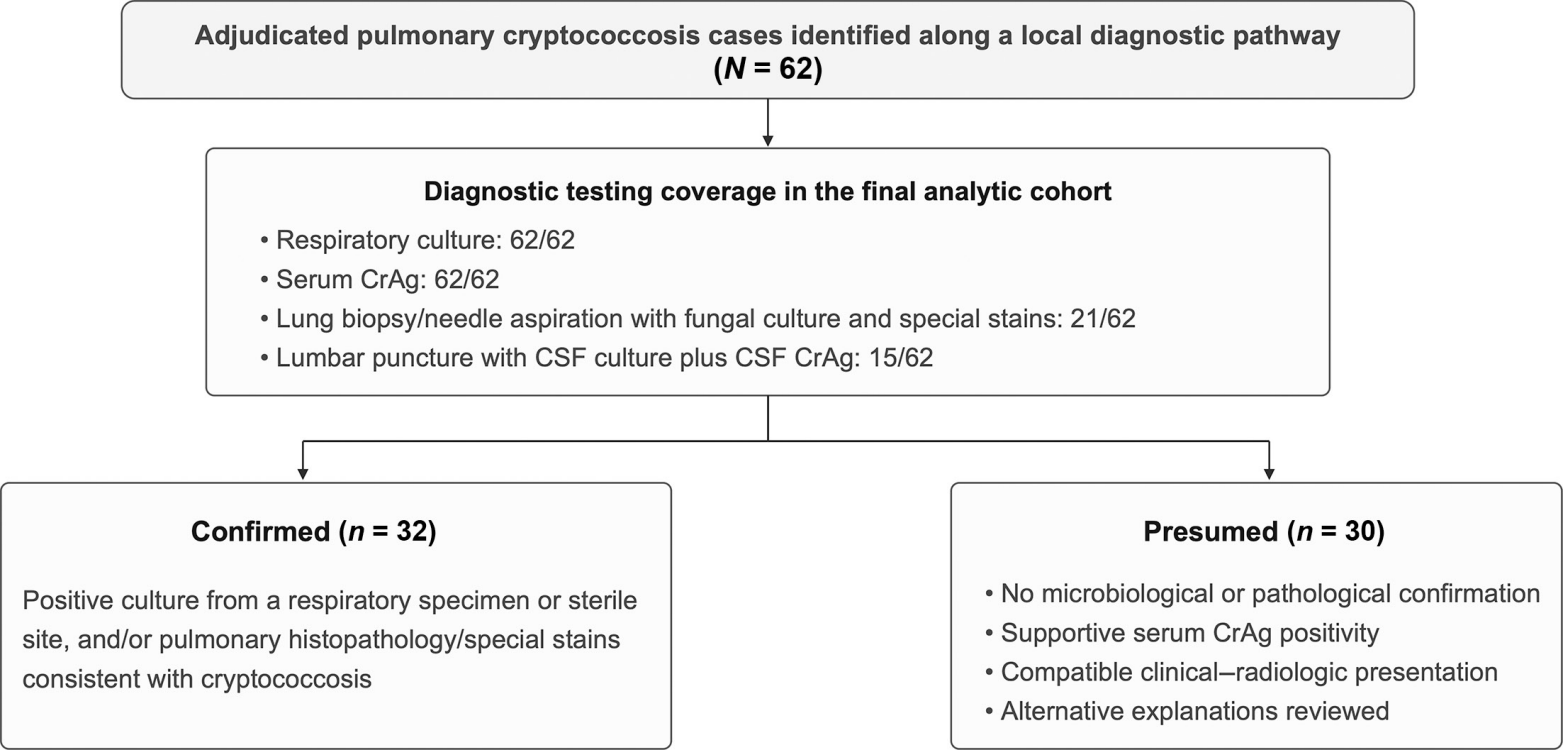
Case inclusion, diagnostic testing coverage and evidence-tier classification (confirmed vs presumed). Cases in the final analytic cohort (*N*=62) were identified along a local routine-care diagnostic pathway among CrAg-tested patients undergoing evaluation for pulmonary imaging abnormalities and were classified into two evidence tiers. Diagnostic testing coverage in the final analytic cohort was as follows: respiratory culture, 62/62; serum CrAg testing, 62/62; lung biopsy/needle aspiration with fungal culture and special stains, 21/62; and lumbar puncture with CSF culture plus CSF CrAg testing, 15/62. Confirmed cases (32/62) were defined by positive culture and/or pulmonary pathology consistent with cryptococcosis. Presumed cases (30/62) lacked microbiological or pathological confirmation but were assigned using a prespecified adjudication framework requiring a compatible clinical–radiologic presentation, supportive serum CrAg positivity and review of available records for major alternative explanations. Because invasive diagnostic procedures were performed selectively in routine care, confirmed and presumed cases are reported separately throughout.

From the screened population, 62 adjudicated pulmonary cryptococcosis cases were included in the final analytic cohort (Fig. 1). Overall, 23/62 (37.1%) were classified as immunocompromised, and 39/62 (62.9%) had no recorded immunocompromising conditions.

The median age was 53.5 years (IQR, 43.3–66.8), including a median of 60.0 (IQR, 51.0–66.5) in the immunocompromised group and 51.0 (IQR, 35.5–66.5) in the no-recorded group (Table 1). Overall, 28/62 (45.2%) were male, including 14/23 (60.9%) in the immunocompromised group and 14/39 (35.9%) in the no-recorded group (Table 1).

**Table 1.** Baseline characteristics of adjudicated pulmonary cryptococcosis cases, overall and by immune status

Characteristic	Overall (*N*=62)	Immunocompromised (*n*=23)	No recorded immunocompromising conditions (*n*=39)
Age, years, median (IQR)	53.5 (43.3–66.8)	60.0 (51.0–66.5)	51.0 (35.5–66.5)
Male sex, *n* (%)	28 (45.2)	14 (60.9)	14 (35.9)
Evidence tier (R1), *n* (%)			
├─ Confirmed	32 (51.6)	10 (43.5)	22 (56.4)
└─ Presumed	30 (48.4)	13 (56.5)	17 (43.6)
Type 2 diabetes mellitus, *n* (%)*	10 (16.1)	10 (43.5)	0 (0.0)
Malignancy, *n* (%)*	4 (6.5)	4 (17.4)	0 (0.0)
Prolonged systemic corticosteroid use, *n* (%)*	4 (6.5)	4 (17.4)	0 (0.0)
Ongoing immunosuppressive therapy, *n* (%)*	3 (4.8)	3 (13.0)	0 (0.0)
HIV infection, *n* (%)*	2 (3.2)	2 (8.7)	0 (0.0)
Cryptococcal meningitis, *n* (%)	11 (17.7)	4 (17.4)	7 (17.9)
Asymptomatic/incidental finding, *n* (%)	16 (25.8)	4 (17.4)	12 (30.8)

*Host factors: Rows under host factors/conditions represent overlapping components of immune vulnerability and therefore do not sum to 100%.

Evidence tier (R1): Confirmed=any positive culture (respiratory or sterile site, including CSF where applicable) and/or histopathology/special stains consistent with cryptococcosis; otherwise, presumed.

Immune status: Immunocompromised=a documented major immunosuppressive condition and/or ongoing immunosuppressive treatment; no recorded immunocompromising conditions=none documented in available records.

HIV, human immunodeficiency virus.

By evidence tier (R1), 32/62 (51.6%) cases were confirmed, and 30/62 (48.4%) were presumed (Fig. 1). Confirmed cases accounted for 10/23 (43.5%) in the immunocompromised group and 22/39 (56.4%) in the no-recorded group (Table 1).

Comorbidities and immunosuppressive exposures were recorded primarily in the immunocompromised group. Type 2 diabetes mellitus was documented in 10/62 (16.1%) overall and in 10/23 (43.5%) of immunocompromised cases. Malignancy, prolonged systemic corticosteroid use, ongoing immunosuppressive therapy and human immunodeficiency virus (HIV) infection were recorded only in the immunocompromised group (Table 1).

### Clinical presentation by immune status group

**Fig. 2.**
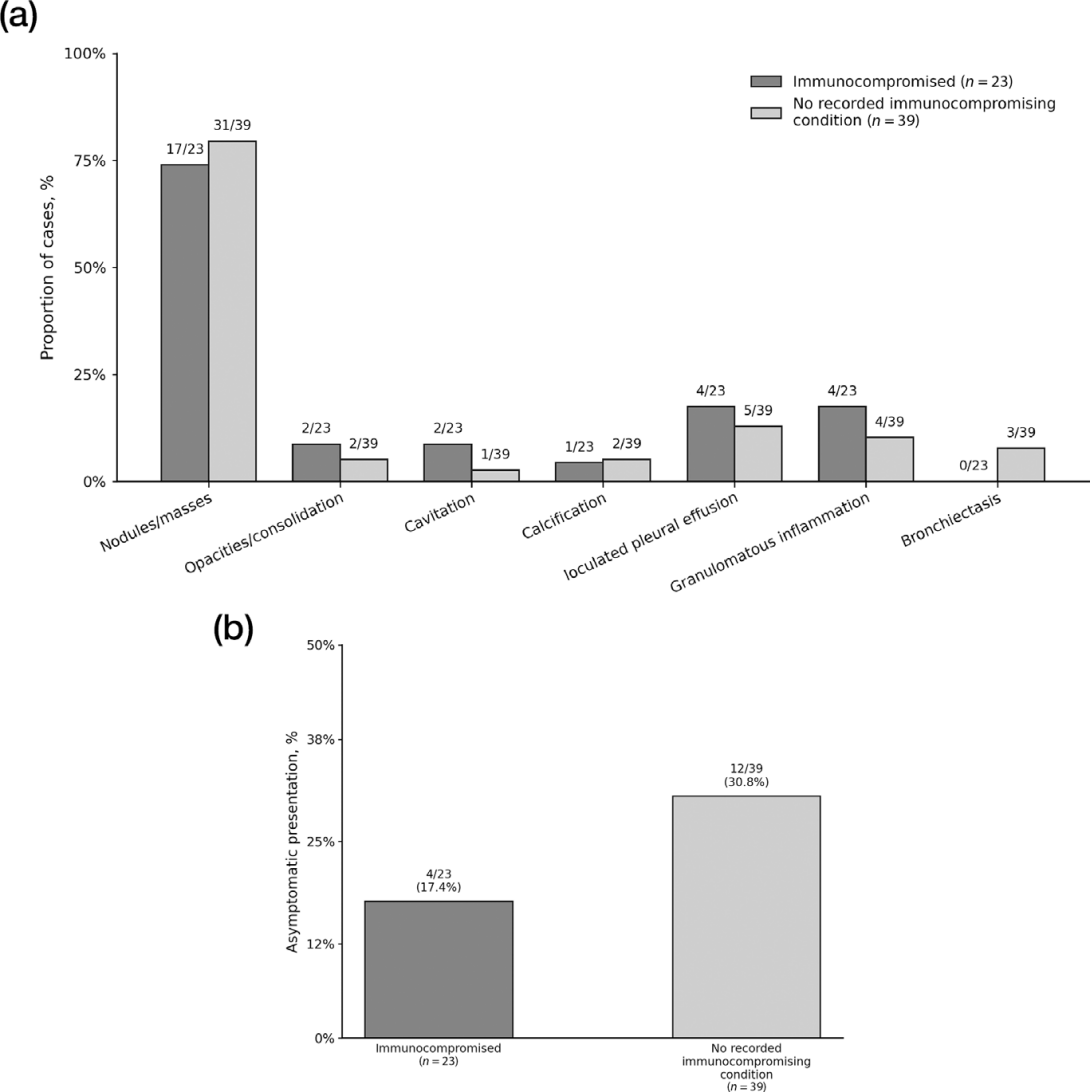
Distribution of chest CT patterns and asymptomatic/incidental presentation by immune status. (**a**) Proportions of key chest CT patterns in immunocompromised cases and cases with no recorded immunocompromising conditions. (**b**) Proportion of asymptomatic/incidental presentation by immune status. Values above bars indicate *n*/*N* (and percentages where shown). Analyses are descriptive only; no hypothesis testing or inferential between-group comparisons were performed.

Asymptomatic/incidental presentation was recorded in 16/62 (25.8%) cases, including 4/23 (17.4%) immunocompromised cases and 12/39 (30.8%) cases with no recorded immunocompromising conditions (Table 1 and Fig. 2b). Symptom profiles by immune status are summarized in Table S1 (*n*/*N*, %).

### Chest CT patterns by immune status group

Chest CT patterns by immune status are summarized in Fig. 2a and Table S2. Nodules/masses were the most frequently recorded pattern in both groups, observed in 17/23 (73.9%) immunocompromised cases and 31/39 (79.5%) cases with no recorded immunocompromising conditions. Opacities/consolidation were recorded in 2/23 (8.7%) and 2/39 (5.1%), respectively. Cavitation was recorded in 2/23 (8.7%) and 1/39 (2.6%), and loculated pleural effusion was recorded in 4/23 (17.4%) and 5/39 (12.8%). Full CT feature distributions are provided in Table S2.

### Evidence tiers (confirmed vs presumed): distribution and key features

Under the prespecified evidence-tier scheme (R1), 32/62 (51.6%) cases were confirmed, and 30/62 (48.4%) were presumed (Fig. 1). Diagnostic testing coverage is reported alongside evidence tiers: respiratory culture and serum CrAg testing were performed in 62/62 cases, lung biopsy/needle aspiration with fungal culture and special stains was performed in 21/62, and lumbar puncture with CSF culture plus CSF CrAg testing was performed in 15/62 (Fig. 1). Evidence tiers are reported separately throughout.

Cryptococcal meningitis was documented in 11/62 (17.7%) cases, including 4/23 (17.4%) in the immunocompromised group and 7/39 (17.9%) in the no-recorded group (Table 1). CNS dissemination by three-level immune strata is summarized descriptively in Fig. S1. A descriptive baseline comparison by pathology-based verification status is provided in Table S4.

### Supplementary descriptive summaries

Symptom profiles and chest CT feature distributions are provided in Tables S1 and S2, respectively. CNS dissemination by three-level immune strata is summarized descriptively in Fig. S1. Re-examination of selected clinical and chest CT patterns in confirmed cases only is provided in Table S3.

## Discussion

A central contribution of this study is methodological transparency in a routine-care diagnostic setting. Rather than treating all cases as equivalent in diagnostic certainty, we prespecified and reported evidence tiers (R1; confirmed vs presumed) and presented them alongside denominators for key diagnostic procedures (culture, serum CrAg and pathology), making work-up intensity and diagnostic certainty explicit. This approach supports interpretable phenotype summaries without using treatment response as diagnostic evidence and may facilitate comparison across routine-care cohorts assembled under different testing pathways and verification practices.

Using routine-care data from a single centre, we describe the clinical and chest CT spectrum of adjudicated pulmonary cryptococcosis cases identified along a local diagnostic pathway among CrAg-tested patients with pulmonary abnormalities, while explicitly separating confirmed from presumed cases under a prespecified evidence-tier scheme. In this cohort of 62 cases, 39 (62.9%) had no recorded immunocompromising conditions, and 23 (37.1%) were immunocompromised. Clinical presentation was often nonspecific, and 16/62 (25.8%) cases were recorded as asymptomatic/incidental findings. On chest CT, nodules/masses were the most frequently recorded pattern in both immune strata (17/23, 73.9% and 31/39, 79.5%). These observations are consistent with prior reports that pulmonary cryptococcosis may be detected during routine evaluation of pulmonary nodules, including in patients with no recorded immunocompromising conditions [1,3].

When summarized by immune status, asymptomatic/incidental presentation was recorded in both strata, including 12/39 (30.8%) cases with no recorded immunocompromising conditions and 4/23 (17.4%) immunocompromised cases, whereas other clinical and CT features showed broadly overlapping descriptive distributions. Cryptococcal meningitis was documented in 11/62 (17.7%) cases and was recorded in both immune strata (4/23, 17.4% and 7/39, 17.9%). Because the present analyses were descriptive and the cohort was assembled through a non-uniform routine-care diagnostic pathway, these findings should be interpreted as observed distributions within this case series rather than as formal between-group differences. In practice, immune status may inform clinical attention, but decisions about CNS assessment should remain guided by symptoms and other clinical findings.

A further strength of this study is the paired reporting of evidence tiers (confirmed vs presumed) and diagnostic testing coverage in a routine-care setting with selective verification. Respiratory culture and serum CrAg testing were available in all 62 cases, whereas pathology-based confirmation was available in 21/62 and CSF-based evaluation in 15/62. We therefore reported confirmed and presumed cases separately rather than collapsing them into a single certainty level. This distinction is particularly relevant in retrospective diagnostic-pathway cohorts, in which invasive diagnostic procedures are not applied uniformly, and confirmed cases are more likely to be identified among patients undergoing higher intensity verification.

These design features also define the main limitations. Because cohort entry depended on serum CrAg testing ordered during evaluation of pulmonary imaging abnormalities, this series reflects adjudicated pulmonary cryptococcosis cases identified along a local diagnostic pathway rather than all pulmonary cryptococcosis cases in the underlying population. Compared with prior multicentre retrospective studies of serum CrAg-based pulmonary cohorts, the present single-centre series is more appropriately interpreted as a pathway-specific descriptive cohort assembled under local ordering and verification practices. The retrospective single-centre design, local ordering practices and non-uniform use of invasive procedures further limit external generalizability, and immune status may be misclassified when documentation is incomplete. Subgroup cell counts, particularly under three-level immune stratification, were small. Some observations may still be relevant across similar routine-care settings, particularly the frequent recording of nodular/mass-like CT presentations and the use of separate evidence-tier reporting when verification is non-uniform. By contrast, the proportion of confirmed cases, the balance between confirmed and presumed cases, and overall cohort composition are likely to remain pathway-dependent. Table S3 provides a confirmed-only re-examination of the main descriptive patterns, and Table S4 summarizes cases by pathology-based verification status.

## Supplementary material

10.1099/jmm.0.002159 Uncited Supplementary Material 1.

## Abbreviations

CNS: central nervous system
CrAg: cryptococcal antigen
CSF: cerebrospinal fluid
CT: computed tomography
HIS: Hospital Information System
HIV: human immunodeficiency virus
IQR: interquartile range
LIS: Laboratory Information System
PACS: Picture Archiving and Communication System
