# Supplementary material for: Pulmonary cryptococcosis in routine care: the clinical–radiologic spectrum and transparent evidence-tier reporting (confirmed versus presumed) among patients with no recorded immunocompromising conditions
Source: J Med Microbiol. 2026 Apr 22;75(4):002159. doi: 10.1099/jmm.0.002159 (PMC13102314; doi:10.1099/jmm.0.002159)
Supplement: Uncited Supplementary Material 1. [file jmm-75-02159-s001.pdf]

Supplementary material

Supplementary Methods S1. Prespecified adjudication checklist for presumed pulmonary cryptococcosis

Presumed pulmonary cryptococcosis was assigned only when all of the following conditions were met:

1. No microbiological or pathological confirmation of cryptococcosis was identified in the available record.
2. A compatible clinical-radiologic pulmonary presentation was present.
3. Serum cryptococcal antigen (CrAg) was positive and used as supportive evidence.
4. Available records were reviewed for major alternative explanations, such as malignancy, tuberculosis, other fungal infection, other pulmonary infection, or noninfectious inflammatory disease.
5. No more plausible alternative diagnosis was retained after clinician adjudication based on the available record.

Treatment response was not used as a standalone diagnostic criterion. Serum CrAg positivity alone was not considered sufficient for presumed-case classification.

Table S1. Clinical presentation and CNS involvement by immune status

A) Respiratory/systemic presentation

Variable	Immunocompromised (n=23)	No recorded immunocompromising condition (n=39)
Cough, n (%)	11 (47.8)	21 (53.4)
Sputum production, n (%)	8 (34.8)	14 (35.9)
Fever, n (%)	7 (30.0)	7 (17.9)
Hemoptysis, n (%)	3 (13.0)	5 (12.8)
Dyspnea, n (%)	3 (13.0)	7 (17.9)
Fatigue, n (%)	2 (8.7)	2 (5.1)
Asymptomatic/incidental finding, n (%)	4 (17.4)	12 (30.8)

B) Neurological symptoms at presentation (not equal to meningitis diagnosis)

Variable	Immunocompromised (n=23)	No recorded immunocompromising condition (n=39)
Headache, n (%)	2 (8.7)	7 (17.9)
Vomiting, n (%)	1 (4.3)	2 (5.1)
Nausea, n (%)	0 (0.0)	1 (2.6)
Meningeal irritation signs, n (%)	0 (0.0)	1 (2.6)
Visual impairment, n (%)	0 (0.0)	1 (2.6)
No neurological symptoms, n (%)	21 (91.3)	33 (84.6)

C) CNS involvement (cryptococcal meningitis; event-level)

Outcome	Immunocompromised (n=23)	No recorded immunocompromising condition (n=39)
Cryptococcal meningitis, n (%)	4 (17.4)	7 (17.9)

Footnote ：

This table is presented descriptively. No prespecified hypothesis testing was performed for individual symptoms due to multiple comparisons and sparse cells.

Table S2. Chest CT patterns by immune status

A) Lesion distribution

Variable	Immunocompromised (n=23)	No recorded immunocompromising condition (n=39)
Bilateral, n (%)	16 (69.6)	26 (66.7)
Left lung only, n (%)	2 (8.7)	6 (15.4)
Right lung only, n (%)	5 (21.7)	7 (17.9)

B) CT patterns and associated findings

Variable	Immunocompromised (n=23)	No recorded immunocompromising condition (n=39)
Nodules/masses, n (%)	17 (73.9)	31 (79.5)
Opacities/consolidation, n (%)	2 (8.7)	2 (5.1)
Cavitation, n (%)	2 (8.7)	1 (2.6)
Calcification, n (%)	1 (4.3)	2 (5.1)
Loculated pleural effusion, n (%)	4 (17.4)	5 (12.8)
Granulomatous inflammation, n (%)	4 (17.4)	4 (10.3)
Bronchiectasis, n (%)	0 (0.0)	3 (7.7)

Footnote

CT features are presented descriptively without inferential testing, given multiple correlated imaging variables and sparse cells in subgroup strata.

Figure S1. Three-level immune strata and CNS dissemination events.

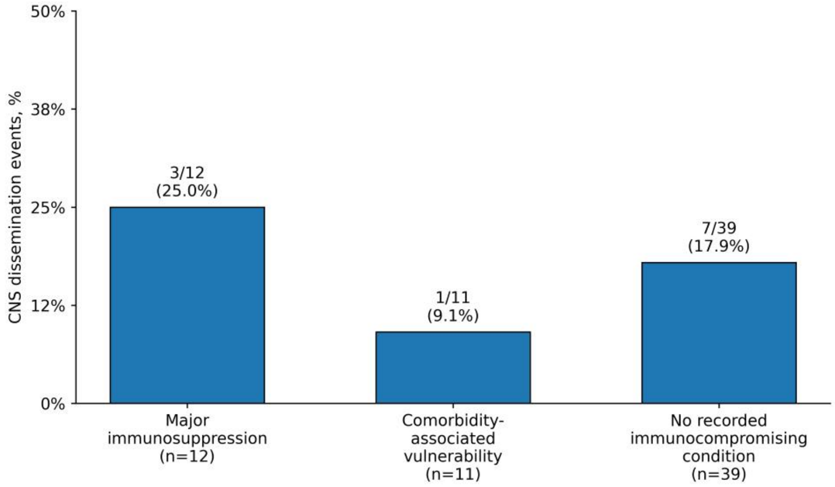

Legend: Bar chart shows the proportion of cases with CNS dissemination events across three immune strata: major immunosuppression, comorbidity-associated vulnerability, and no recorded immunocompromising condition. Values above bars indicate n/N and percentages. Descriptive only; no hypothesis testing or inferential comparisons were performed.

Supplementary Table S3. Descriptive re-examination of selected clinical and chest CT patterns in confirmed cases only

Variable	Confirmed overall (N=32)	Immunocompromised confirmed (n=10)	No recorded immunocompromising condition confirmed (n=22)
Asymptomatic/incidental finding, n (%)	5(15.6)	0(0.0)	5(22.7)
Nodules/masses, n (%)	12 (37.5)	2 (20.0)	10 (45.5)
Opacities/consolidation, n (%)	2 (6.3)	1 (10.0)	1 (4.5)
Cavitation, n (%)	2 (6.3)	1 (10.0)	1 (4.5)
Loculated pleural effusion, n (%)	3 (9.4)	1 (10.0)	2 (9.1)
Cryptococcal meningitis, n (%)	8 (25.0)	4 (40.0)	4 (18.2)

Footnote:

Confirmed cases were defined according to the prespecified evidence-tier classification (R1). This table presents a descriptive re-examination of selected clinical and chest CT patterns restricted to confirmed cases only. Percentages were calculated using the column denominator. No hypothesis testing or inferential between-group comparisons were performed.

Supplementary Table S4. Baseline characteristics by pathology-based verification status

Variable	Underwent pathology-based verification (n=21)	No pathology-based verification (n=41)
Age, years, median (IQR)	52.0 (36.0, 69.0)	54.0 (44.0, 65.0)
Male sex, n (%)	6 (28.6)	22 (53.7)
Immunocompromised, n (%)	5 (23.8)	18 (43.9)
No recorded immunocompromising condition, n (%)	16 (76.2)	23 (56.1)
Confirmed, n (%)	17 (81.0)	15 (36.6)
Presumed, n (%)	4 (19.0)	26 (63.4)
Asymptomatic/incidental finding, n (%)	2 (9.5)	15 (36.6)
Nodules/masses, n (%)	8 (38.1)	23 (56.1)
Opacities/consolidation, n (%)	0 (0.0)	5 (12.2)
Cavitation, n (%)	2 (9.5)	1 (2.4)
Loculated pleural effusion, n (%)	1 (4.8)	2 (4.9)
Cryptococcal meningitis, n (%)	11 (52.4)	0 (0.0)

Footnote:

Pathology-based verification status was defined according to the case-level variable indicating whether pulmonary pathology was performed. This table presents a descriptive baseline comparison by pathology-based verification status to further clarify the potential direction of selective verification. Percentages were calculated using the column denominator. No hypothesis testing or inferential between-group comparisons were performed.
